# West Nile Virus Replication Requires Fatty Acid Synthesis but Is Independent on Phosphatidylinositol-4-Phosphate Lipids

**DOI:** 10.1371/journal.pone.0024970

**Published:** 2011-09-20

**Authors:** Miguel A. Martín-Acebes, Ana-Belén Blázquez, Nereida Jiménez de Oya, Estela Escribano-Romero, Juan-Carlos Saiz

**Affiliations:** Departamento de Biotecnología, Instituto Nacional de Investigación y Tecnología Agraria y Alimentaria (INIA), Madrid, Spain; University of Texas Medical Branch, United States of America

## Abstract

West Nile virus (WNV) is a neurovirulent mosquito-borne flavivirus, which main natural hosts are birds but it also infects equines and humans, among other mammals. As in the case of other plus-stranded RNA viruses, WNV replication is associated to intracellular membrane rearrangements. Based on results obtained with a variety of viruses, different cellular processes have been shown to play important roles on these membrane rearrangements for efficient viral replication. As these processes are related to lipid metabolism, fatty acid synthesis, as well as generation of a specific lipid microenvironment enriched in phosphatidylinositol-4-phosphate (PI4P), has been associated to it in other viral models. In this study, intracellular membrane rearrangements following infection with a highly neurovirulent strain of WNV were addressed by means of electron and confocal microscopy. Infection of WNV, and specifically viral RNA replication, were dependent on fatty acid synthesis, as revealed by the inhibitory effect of cerulenin and C75, two pharmacological inhibitors of fatty acid synthase, a key enzyme of this process. However, WNV infection did not induce redistribution of PI4P lipids, and PI4P did not localize at viral replication complex. Even more, WNV multiplication was not inhibited by the use of the phosphatidylinositol-4-kinase inhibitor PIK93, while infection by the enterovirus Coxsackievirus B5 was reduced. Similar features were found when infection by other flavivirus, the Usutu virus (USUV), was analyzed. These features of WNV replication could help to design specific antiviral approaches against WNV and other related flaviviruses.

## Introduction

West Nile virus (WNV) is a mosquito-borne pathogen responsible for outbreaks of febrile illness, meningitis, encephalitis, and flaccid paralysis. Its main natural hosts are birds, although equines and humans, among other mammals, can also be infected [1]. WNV has been associated with sporadic outbreaks of meningoencephalitis in Africa, Europe, and the Middle East [2]. Since 1999, when the virus emerged for the first time in the USA [3], [4], WNV has spread across the American continent, being responsible of over 30,000 diagnosed infections, more than 12,000 cases of meningitis/encephalitis, and over 1,100 human fatalities [1], [2]. Likewise, since then, over 25,000 accumulated cases in horses have been reported only in the USA [1]. Lately, an increase in the frequency and severity of WNV outbreaks involving equines and humans in Europe and the Mediterranean basin has also been observed [5].

WNV is a plus-strand RNA virus classified within the *Flaviviridae* family inside the genus *Flavivirus* together with other important human pathogens as Dengue virus (DENV), St. Louis encephalitis virus, Yellow Fever virus, or tick-borne encephalitis virus. The *Flaviviridae* family also includes another important human pathogen, the hepatitis C virus, HCV, (*Hepacivirus* genus). As a general feature, cells infected by plus-strand RNA viruses undergo notable intracellular membrane remodelling [6], [7], [8], [9]. For Kunjin virus (KUNV), the Australian variant of WNV, major membrane reorganizations leading to different well defined structures aimed to establish the viral replication complex have been described [6], [10], [11], [12]. The primary membrane source for these structures is provided by the endoplasmic reticulum (ER), although the presence of markers from organelles involved in the endocytic pathway (endosomes/lysosomes) or from the Golgi complex remains unclear [10], [11], [13].

Membrane rearrangements driven by viral infections promote efficient viral replication by achieving the optimal lipid and protein conditions for anchoring viral replication machinery [7]. These phenomena lead to the formation of organelle-like structures specific for virus replication [9], [14]. Regarding lipid composition of these organelle-like structures, a requirement of fatty acid synthesis and the involvement of the key enzyme of this pathway, the fatty acid synthase (FASN), has been documented for enteroviruses (such as poliovirus, PV, and Coxsackievirus B3, CVB3) and members of the *Flavivridae* family [15], [16], [17], [18], [19], thus making of FASN a promising antiviral target. Based on results obtained with CVB3, PV and HCV, it has been also recently proposed that a common specific lipid microenvironment enriched in phosphatidylinositol-4-phosphate (PI4P) is crucial for the replication of RNA viruses [14]. In the case of HCV, this microenvironment was shown to be produced by specific recruitment of the phosphatidylinositol-4-kinase IIIα (PI4KIIIα) and also PI4KIIIβ to the viral replication complex [20], [21], [22], [23], [24]. According to these findings, a number of studies have shown that replication of enteroviruses and HCV is inhibited by the drug PIK93 [14], [24], [25], which specifically blocks the PI4KIIIβ [26] and also interferes with PI4KIIIα [24]. In addition, apart from providing an adequate platform for viral replication, intracellular membrane rearrangements can also favour viral infection by contributing to evade the cellular immune response [27]. In the case of WNV, these membrane rearrangements could play a role for the evasion of innate immune response by interfering with the interferon signalling machinery [28], [29].

Understanding the mechanisms involved in replication complex organization is of crucial interest for the design of novel antiviral approaches. Thus, in the present report we have analyzed the implication of cellular cofactors in membrane rearrangements induced by the highly neurovirulent strain of WNV responsible for the encephalitis outbreak that took place in NY in 1999. These cellular requirements were also investigated for the Usutu virus (USUV), an emerging flavivirus in Europe responsible for recent cases of neuroinvasive disease in humans [30], [31].

## Methods

### Cells, viruses, infections, and virus titration

All manipulations of infectious virus were carried out in Biosafety level 3 (BSL-3) containment facilities. WNV strain NY99 [4], USUV strain SAAR 1776 [32] and CVB5 strain Faulkner [33] were propagated in Vero cells [34]. Vero cells were used in all experiments, except those involving FASN detection, which were performed in Huh-7 cells [35] because commercial antibody tested worked better on this cell line. Procedures for infections have been previously described [34], [36]. Viral titer was determined 24 h postinfection (p.i.) for WNV or USUV and 8 h p.i. for CVB5 by plaque assay. Ten-fold serial dilutions of viral samples were carried out in duplicate and adsorbed to Vero cells grown on six-well tissue culture dishes. After removal of the inoculum, infection was allowed to continue in semi-solid medium containing 1% low-melting-point agarose (Pronadisa, Madrid, Spain) and 2% fetal bovine serum. Infected plates were fixed with 4% formaldehyde at 3 days p.i. Semisolid medium was removed and plaques were visualized by staining with 0.3% crystal violet in 2% formaldehyde plus 10% ethanol.

### Antibodies, stainings and reagents

Double-stranded RNA (dsRNA) and WNV envelope (E) protein were detected using mouse monoclonal antibodies J2 (English & Scientific Consulting Bt., Hungary) and 3.67G (Millipore, Temecula, CA), respectively. Calreticulin and LAMP-1 were detected using Rabbit polyclonal antibodies from Abcam (Cambridge, UK). Rabbit polyclonal antibodies against calnexin and mouse monoclonal antibody against GM130 were from ECM Biosciences (Versailles, KY). Rabbit polyclonal antibody against FASN, and mouse monoclonal anti-α-tubulin B512 were from Sigma (St. Louis, MO). Wheat germ agglutinin (WGA) coupled to Alexa Fluor (AF)-594, To-Pro-3 and secondary antibodies against mouse or Rabbit IgGs coupled to AF-488, -594 or -647 were purchased from Invitrogen (Molecular Probes, Eugene, O). Anti-rabbit and anti-mouse secondary antibodies coupled to horseradish peroxidase were from Dako (Stockholm, Sweden) and Sigma, respectively. PIK93 was from Symansis (Washdyke, New Zeland) and cerulenin and C75 from Sigma. Drugs were dissolved in DMSO before use. Cell viability upon drugs treatments was determined by ATP measurement with CellTiter-Glo® luminescent cell viability assay (Promega, Madison, WI).

### Plasmids and transfections

The following plasmids were used in this study: plasmid IgLdR1kdel encoding an ER targeted mRFP1 [37], plasmids encoding wt forms of Rab4, 5, 7 or 11 fused to GFP [38], [39] and a plasmid encoding GFP-tagged FAPP1-PH [40]. All plasmids were amplified in *Escherichia coli* DH5α, purified using PureLink™ HiPure FP Maxiprep Kit (Invitrogen, Carlsbad, CA) and transfected using FuGENE® HD (Roche, Manheim, Germany) as described by the manufacturer. Cells were infected 24 h post-transfection.

### Electron microscopy

Vero cells grown on 75 cm^2^ tissue culture flasks were infected with WNV or USUV (MOI of 5 PFU/cell) and 24 h p.i. were washed and fixed 30 min at 37°C in 4% paraformaldehyde-2% glutaraldehyde in 0.1 M phosphate buffer pH 7.4 plus 5 mM CaCl_2_. Cells were scrapped and postfixed in 1% osmium tetroxide-1% potassium ferricyanide for 1 h at 4°C, washed three times with bidistilled water and treated with 0.15% tanic acid (1 min). Cells were washed with the buffer and with bidistilled water prior to the staining with 2% uranyl acetate (1 h). After three washes with bidistilled water samples were dehydrated in ethanol and embedded in the resin. Samples were examined using a Jeol JEM-1010 electron microscope (Jeol, Japan) operated at 80 kV and images were acquired using a digital camera 4 K×4 K TemCam-F416 (Tietz Video and Image Processing Systems GmbH, Gauting, Germany).

### Immunofluorescence and confocal microscopy

Following 24 h of infection, cells grown on glass coverslips (Menzel-Glaser, Braunschweig, Germany) were washed twice with phosphate buffer saline (PBS) and fixed in 4% paraformaldehyde in PBS for 15 min at room temperature (RT). Samples were washed twice in PBS and blocked, and permeabilized in BPTG (1% bovine serum albumin [BSA], 0.1% Triton-X 100, 1 M glycine in PBS) for 15 min at room temperature (RT). Then, cells were incubated (1 h at RT) with primary antibodies diluted in 1% BSA in PBS. After two additional washes with PBS, cells were incubated (30 min at RT) with fluorescently labelled secondary antibodies or WGA coupled to AF594. Nuclei were stained using To-Pro-3 as described by the manufacturer. Samples were washed again with PBS and mounted in Fluoromount-G™ (Southern Biotech, Birmingham, AL). Cells were observed using a Leica TCS SPE confocal laser scanning microscope using an HCX PL APO 63×/1.4 oil immersion objective. Images were acquired using Leica Advanced Fluorescence Software and processed with ImageJ (http://rsbweb.nih.gov/ij/) and Adobe Photoshop CS2 (Adobe Inc, San Jose, CA). Optical slice thickness for all confocal images displayed was of 1 airy unit.

### Quantitative RT-PCR

Infected cultures were subjected to two freeze and thaw cycles and viral RNA was extracted with NucleoSpin viral RNA isolation kit (Macherey-Nagel, Düren, Germany) at 24 h p.i. from samples treated with the drugs from 3 h p.i.. The amount of viral RNA copies was determined by quantitative RT-PCR [41] as genomic equivalents to PFU/ml by comparison with RNA extracted from previously titrated samples [42], [43].

### Western blot

Cells were lysed on ice in RIPA buffer (150 mM NaCl, 5 mM β-mercaptoethanol, 1% NP-40, 0.1% sodium dodecyl sulfate [SDS], 50 mM Tris-HCl pH 8) supplemented with cOmplete protease inhibitor cocktail tablets (Roche) and protein concentration was determined by Bradford assay. Equal amounts of proteins were mixed with Laemmli sample buffer, subjected to SDS-PAGE and proteins were electrotransferred onto a nitrocellulose membrane. Membrane was blocked with 5% skimmed milk in PBS 0.05% Tween-20, incubated with primary antibodies (over night at 4°C), washed three times with the same buffer, and subsequently incubated with secondary antibodies coupled to horseradish peroxidase (1 h at RT) diluted in 1% skimmed milk in PBS-Tween. Membrane was washed three times and proteins were detected by chemiluminiscence using a ChemiDoc™ XRS+ System (Bio-Rad, Hercules, CA).

### Data analysis

Analysis of the variance (ANOVA) was performed with the statistical package SPSS 15 (SPSS Inc., Chicago, IL) applying Bonferroni's correction for multiple comparisons. Data are presented as means ± standard deviations. Asterisks (*) in the figures denote statistically significant differences (*P*<0.05).

## Results

### Intracellular membrane rearrangements involved in WNV replication complex assembly

**Figure 1:**
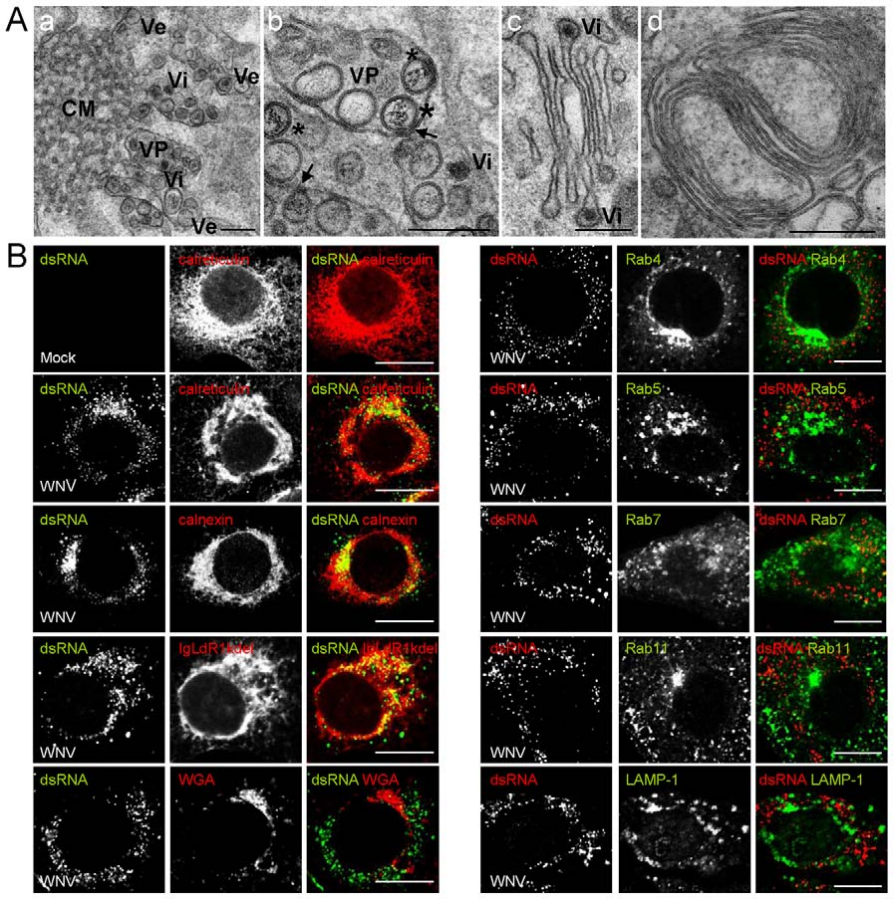
Analysis of cellular components involved in WNV replication complex. (A) Ultrastructure of WNV-induced membrane alterations. Cells infected with WNV (MOI of 5 PFU/cell) were fixed and processed for electron microscopy at 24 h p.i. (a) Electron micrograph showing membrane alteration on WNV infected cells: convoluted membranes (CM), WNV induced-vesicles (Ve), vesicle packets (VP), and electron dense virions (Vi). (b) Higher magnification images of VP induced by WNV infection. Black arrows indicate the point of contact between a vesicle and the outer membrane of the VP. Asterisks denote Ve with electron dense fibrous material. (c) WNV virions trafficking through the Golgi complex. (d) Whorls of stacked membranes. Scale bars: 200 nm. (B) Cells infected or not (mock) as in (A) were fixed and processed for immunofluorescence and confocal microscopy. WNV dsRNA was detected using a monoclonal antibody and cellular structures were labelled by using specific antibodies, or by transfection with plasmids encoding fluorescent fusion proteins (see text for details). Suitable secondary antibodies coupled to AF488 or 594 were used. Scale bar: 10 µm.

When intracellular membrane rearrangements in WNV-NY99 infected cells (24 h p.i.) were analyzed by transmission electron microscopy, convoluted membranes (CM) and vesicle packets (VP) were observed (Figure 1A panels a and b). These VPs contained electron dense virions (Vi) and spherical vesicles (Ve), some of which presented electron dense fibrous material inside (Figure 1A, panel b asterisks). In some cases, association of these vesicles to the external membrane of VP was observed (Figure 1A, panel b arrows). Electron dense virions located at the tip of the cisternae inside Golgi-like structures were also observed (Figure 1A, panel c). The localization of viral particles at the Golgi complex was supported by colocalization of the structural envelope (E) protein of the virions with this organelle (Figure S1). In addition to these membrane rearrangements, whorls of stacked ribosome free membranes were also observed in infected cells (Figure 1A, panel d).

By means of immunofluorescence and confocal microscopy, cell structures were visualized using either specific antibodies against cellular markers (calnexin and calreticulin for ER, and LAMP-1 for lysosomes), and wheat germ agglutinin (WGA) as a marker of the Golgi complex, or by performing transfections with plasmids encoding fluorescent proteins markers for different cellular compartments (IgLdR1kdel for ER, and Rab 4, 5, 7, and 11 for endosomes) (Figure 1B). Signal from double-stranded RNA (dsRNA), a marker of the viral replication complex, was observed as cytoplasmic foci in infected cells, but not in mock-infected cells. However, WNV dsRNA only colocalized with markers from the ER (calnexin, calreticulin and IgLdR1kdel), and excluded the WGA marker from Golgi complex, as well as markers from early, recycling and late endosomes (Rab 4, 5, 7 and 11) or lysosomes (LAMP-1).

### WNV replication requires fatty acid synthesis

**Figure 2:**
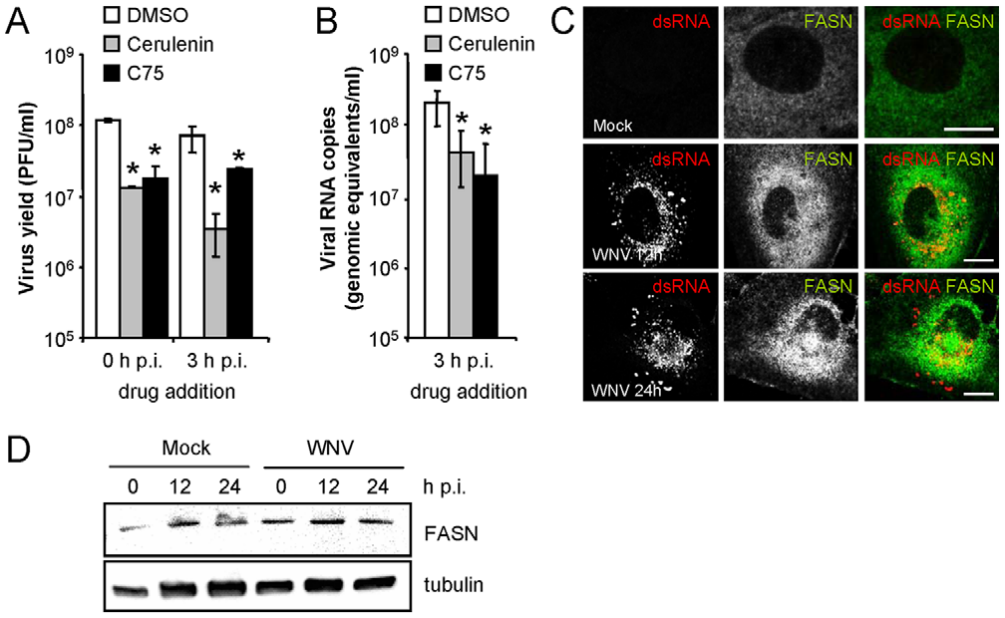
Replication of WNV is dependent on fatty acid synthesis. (A) WNV infection requires active fatty acid synthesis. Cells infected with WNV (MOI of 0.5 PFU/cell) were treated with 15 µM cerulenin or 15 µM C75 from 0 or 3 h p.i. throughout the rest of the assay and total virus yield was determined at 24 h p.i. (B) Genome replication of WNV is dependent on fatty acid synthesis. Cells were infected and treated with FASN inhibitors from 3 h p.i. as in (A). RNA was extracted at 24 h p.i. and the number of WNV RNA copies was determined by quantitative RT-PCR. (C) Localization of FASN in mock and WNV-infected Huh-7 cells. Infected cells (MOI of 5 PFU/cell) were fixed and processed for immunofluorescence (12 or 24 h p.i.) using a rabbit anti-FASN antibody in combination with a monoclonal antibody against dsRNA. Primary antibodies were detected using suitable AF-488 or 594 labelled secondary antibodies. Scale bar: 10 µm. (D) Analysis of FASN levels during WNV infection. Huh-7 cells were infected with WNV as in (A) and lysed at different times p.i. Western blot analysis was performed to determine the relative levels of FASN protein. Membrane was retested against a tubulin antibody as a control for protein loading.

As WNV replication induced notable intracellular membrane rearrangements, the involvement of different cellular lipids on WNV replication was analyzed. The role of fatty acid synthesis in WNV replication was analyzed by using two pharmacological inhibitors of FASN: cerulenin and C75 [44]. Both cerulenin and C75 reduced WNV production when added to the culture medium either at 0 h or 3 h p.i. (Figure 2A), thus showing that these drugs affected viral replication stages rather than entry steps. Working concentrations of these inhibitors had no major effects on cell viability (Figure S2), indicating that the inhibition of viral production could be attributed to the inhibition of the targeted cellular process. Viral replication in the presence of FASN inhibitors was further investigated analyzing viral RNA synthesis by means of quantitative RT-PCR (Figure 2B). These experiments confirmed that inhibition of WNV production by cerulenin and C75 when added 3 h p.i. was derived from a reduction in the synthesis of viral RNA. Then, the location of FASN in WNV-infected cells was analyzed by inmunofluorescence microscopy (Figure 2C). Mock-infected cells displayed a diffuse cytoplasmic staining of FASN, whereas localization of FASN close to WNV replication complexes was observed in infected cells at 12 or 24 h p.i. Analysis by western blot revealed that no significant changes in the relative levels of FASN were observed during WNV infection (Figure 2D).

### WNV replication is independent of PI4P

**Figure 3:**
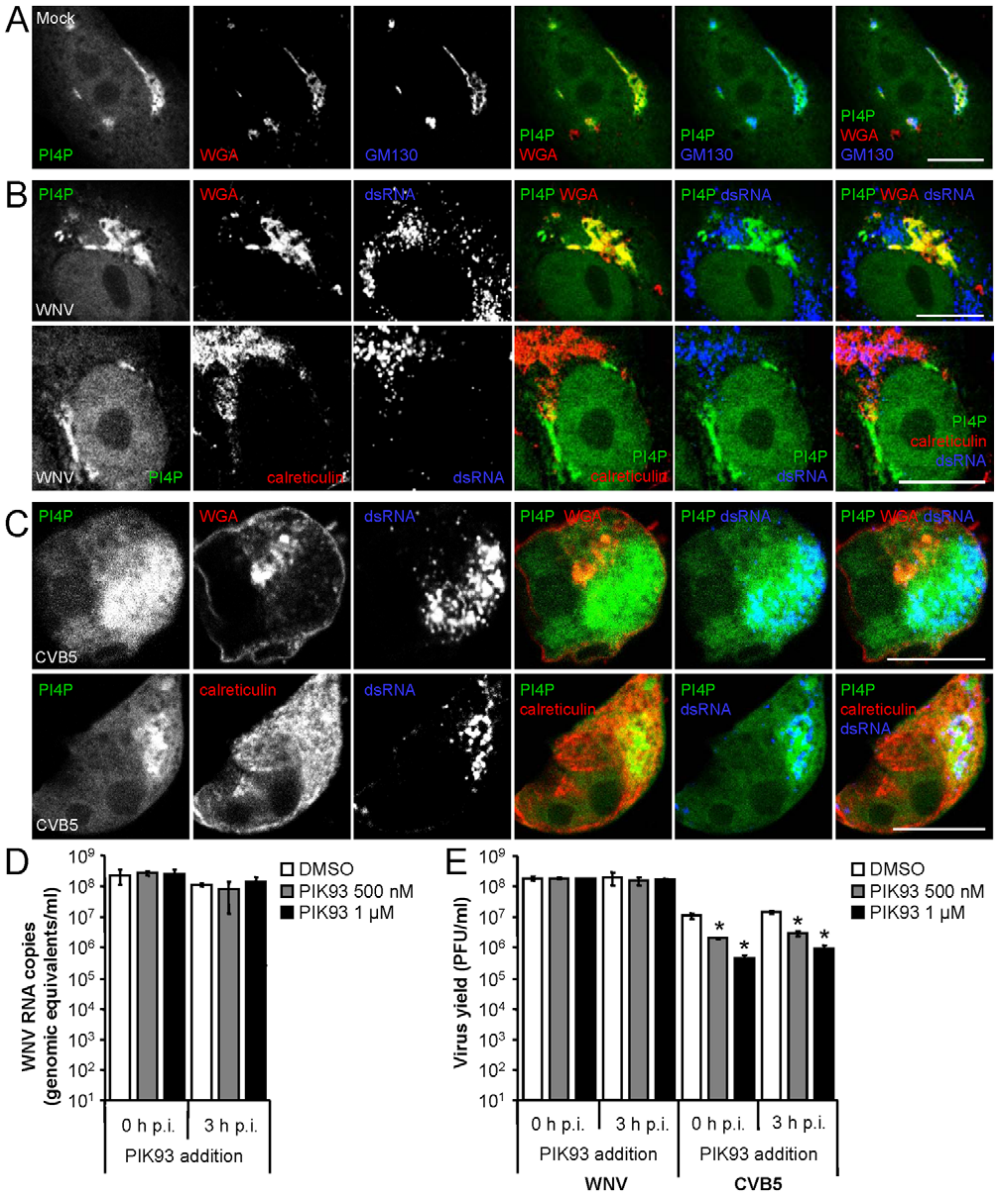
Replication of WNV is independent of PI4P. (A) Localization of PI4P at the Golgi complex in mock-infected cells. Cells transfected with a plasmid encoding a GFP-tagged FAPP1-PH protein to detect PI4P were fixed and processed for immunofluorescence (24 h p.i.) using WGA-AF594 and a mouse monoclonal antibody against GM130 (revealed with a secondary antibody coupled to AF647). (B) Localization of PI4P in WNV infected cells. Cells transfected as in (A) and later infected with WNV (MOI of 5 PFU/cell) were fixed and processed for immunofluorescence (24 h p.i.). WGA labelled with AF594 or a rabbit anti-calreticulin antibody was used in combination with a monoclonal antibody against dsRNA. Primary antibodies were detected using suitable AF594 or 647 labelled secondary antibodies. (C) Cells transfected as in (A) and later infected with CVB5 (MOI of 5 PFU/cell) were fixed and processed for immunofluorescence (8 h p.i.) as described in (B). (D) WNV RNA replication is independent of PI4KIIIβ function. Cells infected with WNV (MOI of 0.5 PFU/cell) were treated with different concentrations of PIK93 from 0 or 3 h p.i. throughout the rest of the assay. RNA from infected plates was extracted at 24 h p.i. and the number of WNV RNA copies was determined by quantitative RT-PCR. (E) Cells were infected with WNV or CVB5 and treated with PIK93 as in (D). Total virus yield (24 h p.i. for WNV and 8 h p.i. for CVB5) was determined by plaque assay. Scale bars: 10 µm.

The role of PI4P lipids, which have been shown to play an important role in RNA virus replication (see Introduction), was analyzed on WNV replication. As shown in Figure 3A, in mock-infected cells transfected with a plasmid encoding a GFP-tagged FAPP1-PH protein, which binds to PI4P lipids, PI4P localized at discrete structures surrounding cell nuclei, colocalizing with Golgi markers WGA and GM130, and thus indicating a primary distribution of this lipid at the Golgi complex. Likewise, confocal laser scanning microscopy of cells transfected with PI4P reporter plasmid and later infected with WNV did not show redistribution of PI4P, and colocalization of PI4P with WGA close to the nucleus was observed, thus indicating that PI4P was also located at the Golgi complex in WNV infected cells (Figure 3B). Even more, no colocalization of PI4P with dsRNA was observed (Figure 3B upper panels), since when calreticulin was used as a marker for the ER it was noted that dsRNA was located at the ER and excluded PI4P lipids (Figure 3B lower panels). On the other hand, enterovirus CVB5-infected cells showed a redistribution of PI4P lipids that could be also found outside Golgi complex (Figure 3C upper panels) and not excluding the ER (Figure 3C lower panels). In addition to these, PI4P lipids colocalized with dsRNA, showing that they were located to CVB5 replication complexes (Figure 3C). Next, the effect of the inhibition of PI4P synthesis by PIK93 was analyzed. To this end, when WNV replication was monitored by quantitative RT-PCR and virus titration (24 h p.i.), no statistically significant differences on WNV RNA synthesis (Figure 3D) and no reduction on viral production were observed upon PIK93 treatment (Figure 3E) when the drug was added at 0 or 3 h p.i. The capability of PIK93 to interfere on viral replication was confirmed by the effect that it exerted on CVB5 infected cells, in which a dose-dependent inhibition was observed when PIK93 was added either at 0 or 3 h p.i.

### Replication of USUV shares common requirements with WNV

**Figure 4:**
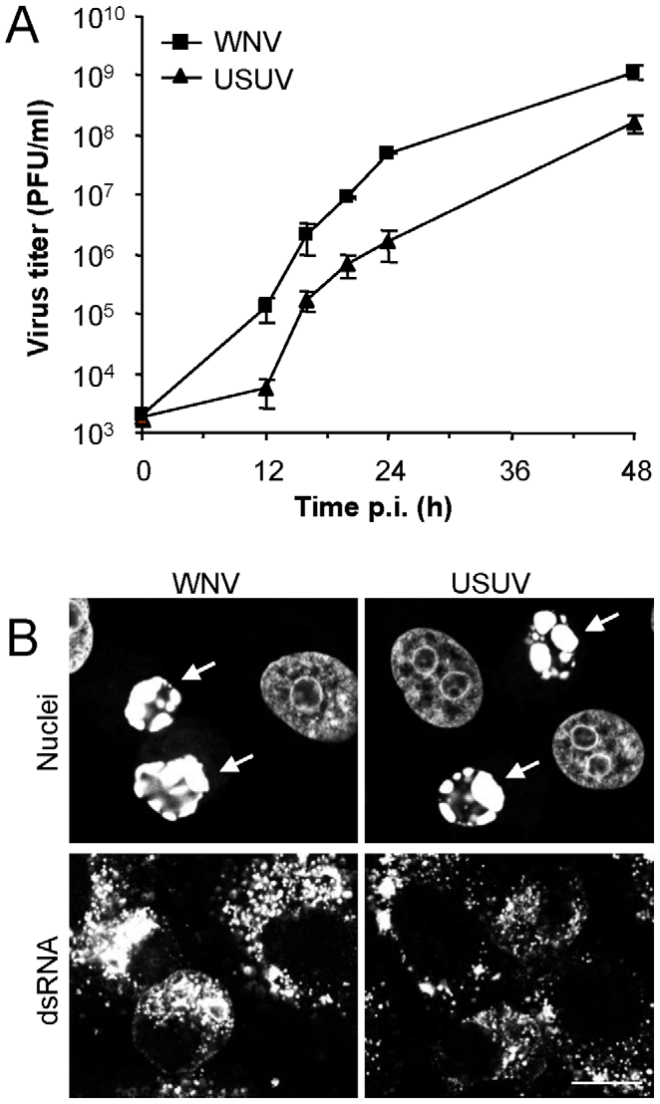
Comparative analysis of WNV and USUV infection and cytophatology in Vero cells. (A) Growth curve of WNV and USUV. Cells were infected (MOI of 0.1 PFU/cell) and supernatant virus yield was determined at different times p.i. (B) Cells were infected as in (A), fixed and processed for immunofluorescence and confocal microscopy at 48 h p.i. Monoclonal antibody against dsRNA and AF-488 labelled secondary antibodies were used to detect dsRNA. Cell nucleus was stained with To-Pro3. Arrows point to apoptotic cell nuclei. Scale bar: 10 µm.

**Figure 5:**
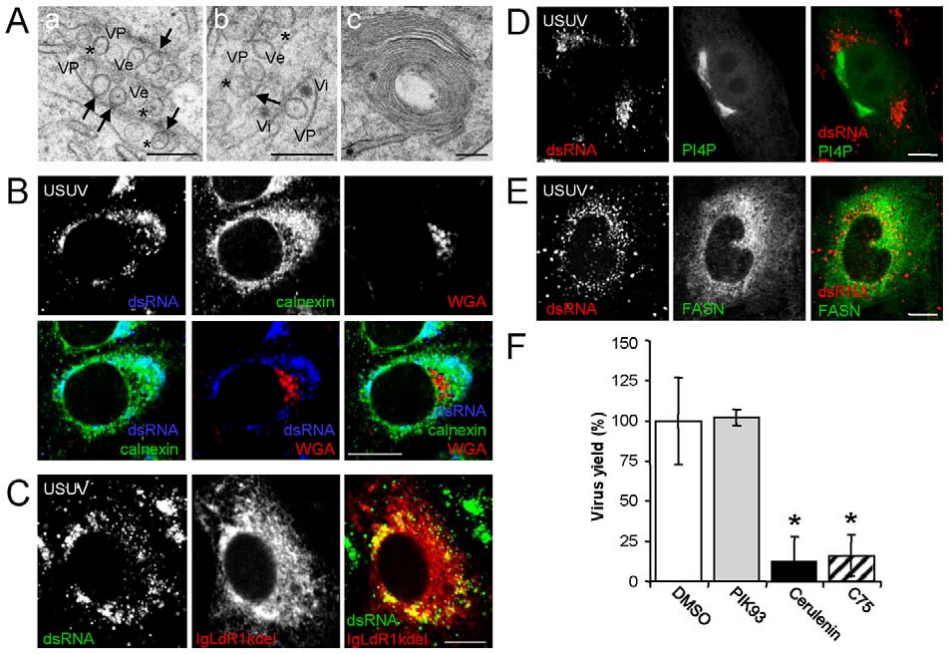
Replication of USUV is associated to the ER, requires fatty acid synthesis and is independent of PI4P. (A) Ultrastructure of USUV-induced membrane alterations. Vero cells infected with USUV (MOI of 5 PFU/cell) were fixed and processed for electron microscopy at 24 h p.i. (a) and (b) Electron micrographs showing membrane alteration on USUV infected cells: induced-vesicles (Ve), vesicle packets (VP), and electron dense virions (Vi). Black arrows indicate the point of contact between a vesicle and the outer membrane of the VP. Asterisks denote Ve with electron dense fibrous material. (c) Whorls of stacked membranes. Scale bars: 200 nm. (B). Cells infected as in (A) were fixed and processed for immunofluorescence using a rabbit anti-calnexin antibody combined with a monoclonal antibody against dsRNA and WGA labelled with AF594. (C) Cells transfected with plasmid encoding an mRFP1 version coupled to an ER retention signal (IgLdR1kdel) were infected with USUV as is (A) and then fixed and processed for immunofluorescence using a monoclonal antibody against dsRNA. (D) Cells transfected with plasmid encoding a GFP-tagged FAPP1-PH protein (to detect PI4P) were infected with USUV as is (A) and then fixed and processed for immunofluorescence using a monoclonal antibody against dsRNA (E) USUV-infected Huh-7 cells were stained using rabbit anti-FASN antibodies combined with a monoclonal antibody against dsRNA. Suitable secondary antibodies coupled to AF-488, 594 or 647 were used in (B), (C), (D) and (E). (F) Vero cells infected with USUV (MOI of 0.5 PFU/cell) were treated with 1 µM PIK93, 15 µM cerulenin, or 15 µM C75 from 3 h p.i. throughout the rest of the assay, and total virus yield (24 h p.i.) was determined by standard titration in semisolid medium. Scale bars: 10 µm.

The findings observed for WNV replication were also investigated using another *Flavivirus* of the Japanese encephalitis serocomplex. Infection with USUV in Vero cells cursed with cythopathic effect, and shared comparable growth kinetics to that of WNV although USUV titers were one order of magnitude lower (Figure 4A). Infection by both viruses induced apoptosis in cultured cells at late infection stages, since cell nuclei displaying apoptotic characteristics, as chromatin condensation and marginalization, were observed (Figure 4B, arrows). Cells infected with USUV (24 h p.i.) were analyzed by transmission electron microscopy (Figure 5A). Vesicle packets (VP) were observed (Figure 1A panels a and b). As commented for WNV, these VPs contained electron dense virions (Vi) and spherical vesicles (Ve), some of which presented electron dense fibrous material inside (Figure 1A, panels a and b asterisks). Association of these vesicles to the external membrane of VP was also noted (Figure 1A, panels a and b arrows). Whorls of stacked ribosome free membranes were also observed (Figure 1A, panel c). Next, cells infected with USUV were immunostained and analyzed by confocal microscopy. As described for WNV, in USUV-infected cells dsRNA colocalized with calnexin (an ER marker), but excluded WGA signal, a Golgi marker (Figure 5B). Association of USUV dsRNA with ER was also observed in infected cells that had been previously transfected with IgLdR1kdel plasmid, an ER reporter (Figure 5C), confirming that USUV replication also took place associated to ER. When lipid requirements for USUV replication were analyzed, cells transfected with PI4P reporter plasmid and infected with USUV did not show association of PI4P to viral replication complex (Figure 5D), although association of FASN to viral replication complex was observed (Figure 5E). In addition to this, infection by USUV was not inhibited by PIK93, whereas virus yield was reduced when cerulenin or C75 were added to the cultures 3 h p.i., thus confirming fatty acid requirements instead of PI4P for USUV replication (Figure 5F).

## Discussion

Recent advances indicate that *Flavivirus* replication complex is constituted upon structures derived from the ER [13], [45]. According to this observations, the internal ‘vesicles’ observed inside VPs, which contain dsRNA [13], [45], actually constitute invaginations from the external membrane and contact by pores with the cell cytoplasm [13], [45]. Supporting this model, we have observed an association of these vesicles to the external membrane of the VP, and a fibrous material stained by uranyl acetate, which binds to phosphate groups present in nucleic acids [45], was also observed inside some of these structures. Assembled virions may bud into the ER (which were also observed in VP packets together with vesicles), and traffic across the Golgi complex for maturation, prior to extracellular release [6], [10], [11]. In addition to VPs, CMs, which may be derived from the rough ER [11], as well as whorls of stacked ribosome free membranes similar to organized smooth ER structures [46], [47] were observed. Supporting the origin of these membranes from ER, dsRNA only colocalized with markers from the ER. Regarding assembly of the viral replication complex among different members of the *Flaviviridae* family, HCV replication complex also contains components of the endocytic machinery normally associated to endosomes, i.e. Rab 4, 5 and 7 [23], [48], and Rab 7L1 has been also associated with DENV replication [16]. Our results showed that all markers of endocytic organelles tested, Rab 4, 5, 7, 11 and LAMP-1, which included early and late endosomes as well as lysosomes, were not associated to dsRNA in WNV infected cells, thus indicating that endocytic machinery components are not recruited to membranous structures where WNV replication takes place.

Membrane rearrangements driven by viral replication are connected with lipid metabolism [14], [18], [19], [49]. In this way, the specific lipid content of membranes has been related either to the achievement of proper membrane fluidity, plasticity and topology (helping membrane curvature) or to favouring the recruitment of viral and cellular factors to the replication complex [14], [50], [51], [52]. Flaviviruses manipulate host-cell machinery to create an optimal specific lipid microenvironment for assembly of their replication complex, where cholesterol seems to play an essential role [28], [53]. In this study lipid requirements, other than cholesterol, in WNV replication were addressed. Fatty acid synthesis was found to be related to WNV replication, as RNA replication and virus production were reduced by drugs targeting this process. These findings are consistent with previous reports analyzing the involvement of fatty acid synthesis and FASN activity on the replication of virus of the *Flaviviridae* family [16], [17]. In the case of DENV, it has been documented that FASN is recruited to the viral replication complex by specific interaction with viral protein [16]. Regarding FASN levels, they were not substantially altered by WNV infection when analyzed by western blot, consistent with that reported for DENV [16] and in contrast to HCV-infection, which curses with an increase in FASN expression [17]. The involvement of fatty acid synthesis in viral replication seems to be a widely used strategy for RNA virus replication, since enzymes of the fatty acid metabolic pathway are also involved in intracellular membrane remodelling of a variety RNA virus [49], [52], [54].

It has been also recently proposed that a common specific lipid microenvironment specifically enriched in PI4P is crucial for the replication of RNA viruses as enteroviruses (CVB3 and PV) and HCV [14]. PI4P location to the viral replication complex is mediated by recruitment of a lipid kinase that synthesizes this lipid, namely PI4KIIIα for HCV and PI4KIIIβ for CVB3, PV, and also HCV [14], [20], [21], [22], [23], [24], [25]. Our results show that, in contrast to what has been described for CVB3 and HCV [14], [22], and observed in this report for CVB5, PI4P was not redistributed in WNV infected cells. Even more, the fact that PI4P lipids did not colocalize with dsRNA indicates that they were not located at WNV replication complex, opposite to that observed for CVB5. In addition to these, when PIK93, a drug that specifically blocks the PI4KIIIβ [26], although it can also inhibit PI4KIIIα [24], was used, it was observed that concentrations of PIK93 that successfully inhibited replication of enteroviruses and HCV [14], [24], [25] did not inhibit infection with WNV, or WNV RNA synthesis. This lack of PIK93 effect revealed an independence of PI4P for WNV and USUV replication, in contrast to that previously suggested for enteroviruses and HCV replication [14], [24].

As it has been suggested that comparative studies using different flaviviruses may unlock crucial mechanisms of disease pathogenesis [27], similar experiments were carried out using USUV. Supporting WNV results, USUV-infected cells displayed similar membrane rearrangements to that induced by WNV, and dsRNA also was localized at the ER. When lipid requirements for USUV infection were analyzed, no PI4P relocalization or colocalization with dsRNA was observed, but USUV infection, as well as WNV infection, was also sensitive to inhibition of fatty acid synthesis and was not affected by PIK93.

Modulation of lipid composition, specially cholesterol, is related to viral-induced membrane rearrangements [47]. Along this line, fatty acid biosynthesis may also act in concert with cholesterol synthesis to enable proper membrane rearrangements for replication complex assembly [16], [55]. In addition to this, a link between fatty acid requirements for virus replication and the induction of autophagy, as an alternative source of fatty acids, has been also recently reported for DENV infection [56]. The whorls of stacked ribosome free membranes observed in this study in WNV and USUV infected cells resembled multi-lamellar bodies, which have been reported to be associated with autophagy, and whose formation is also regulated through lipid composition [57], [58]. Although authophagy has been characterized for other members of the *Flaviviridae* family as DENV, HCV, and Modoc virus [56], [59], [60], in the case of WNV and USUV the potential role of autophagy in viral replication remains to be explored. However, it has been recently reported that infection by WNV results in an induction of the unfolded protein response [61], [62], which, in other viral models leads to the induction of autophagy [63].

In summary, our observations indicate that WNV replication complex, organized by remodelling membranes derived from the ER, is dependent on fatty acid synthesis but does not share some common features described for other members of the same family (*Flaviviridae*), i.e. PI4P requirements or involvement of the endocytic machinery. Apart from providing basic information of the cellular mechanisms involved in flaviviral replication, these unique features of WNV and USUV replication may help in the design of specific antiviral approaches.

## Supporting Information

Figure S1 **Localization of WNV E protein at the Golgi complex.** Vero cells infected with WNV (MOI of 5 PFU/cell) were fixed and processed for immunofluorescence (24 h p.i.) using a monoclonal antibody against E glycoprotein revealed with a suitable AF488 coupled secondary antibody, and WGA lectin AF594 as a Golgi marker. Scale bar: 10 µm.
(TIF)

Figure S2 **Analysis of cellular viability upon drug treatments.** Cellular ATP levels were determined after 24 h of treatment with DMSO (drug vehicle), 15 µM cerulenin, 15 µM C75 or 1 µM PIK93. RLU, relative luciferase units.
(TIF)
