# Supplementary figures and images for: West Nile Virus Replication Requires Fatty Acid Synthesis but Is Independent on Phosphatidylinositol-4-Phosphate Lipids
Source: PLoS One. 2011 Sep 20;6(9):e24970. doi: 10.1371/journal.pone.0024970 (PMC3176790; doi:10.1371/journal.pone.0024970)

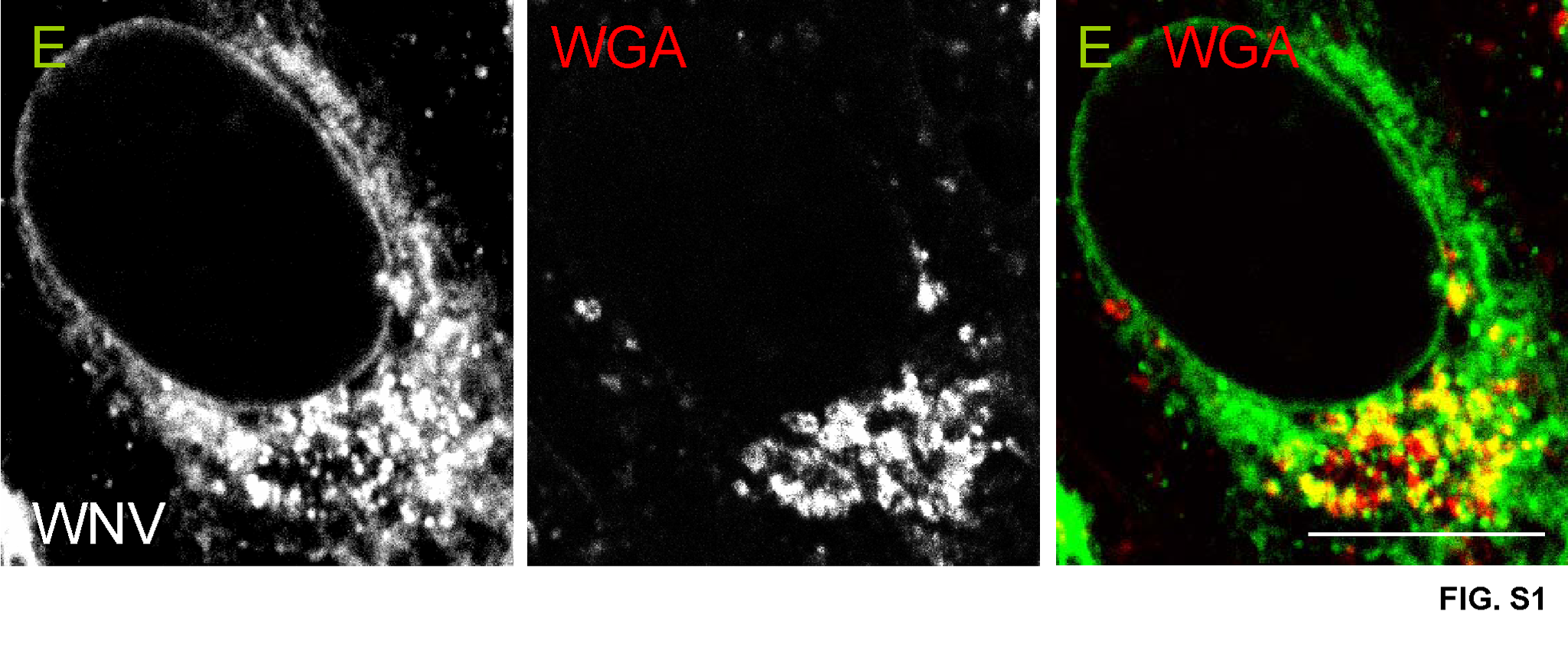

Supplement: Figure S1 — Localization of WNV E protein at the Golgi complex. Vero cells infected with WNV (MOI of 5 PFU/cell) were fixed and processed for immunofluorescence (24 h p.i.) using a monoclonal antibody against E glycoprotein revealed with a suitable AF488 coupled secondary antibody, and WGA lectin AF594 as a Golgi marker. Scale bar: 10 µm. (TIF) [file pone.0024970.s001.tif]

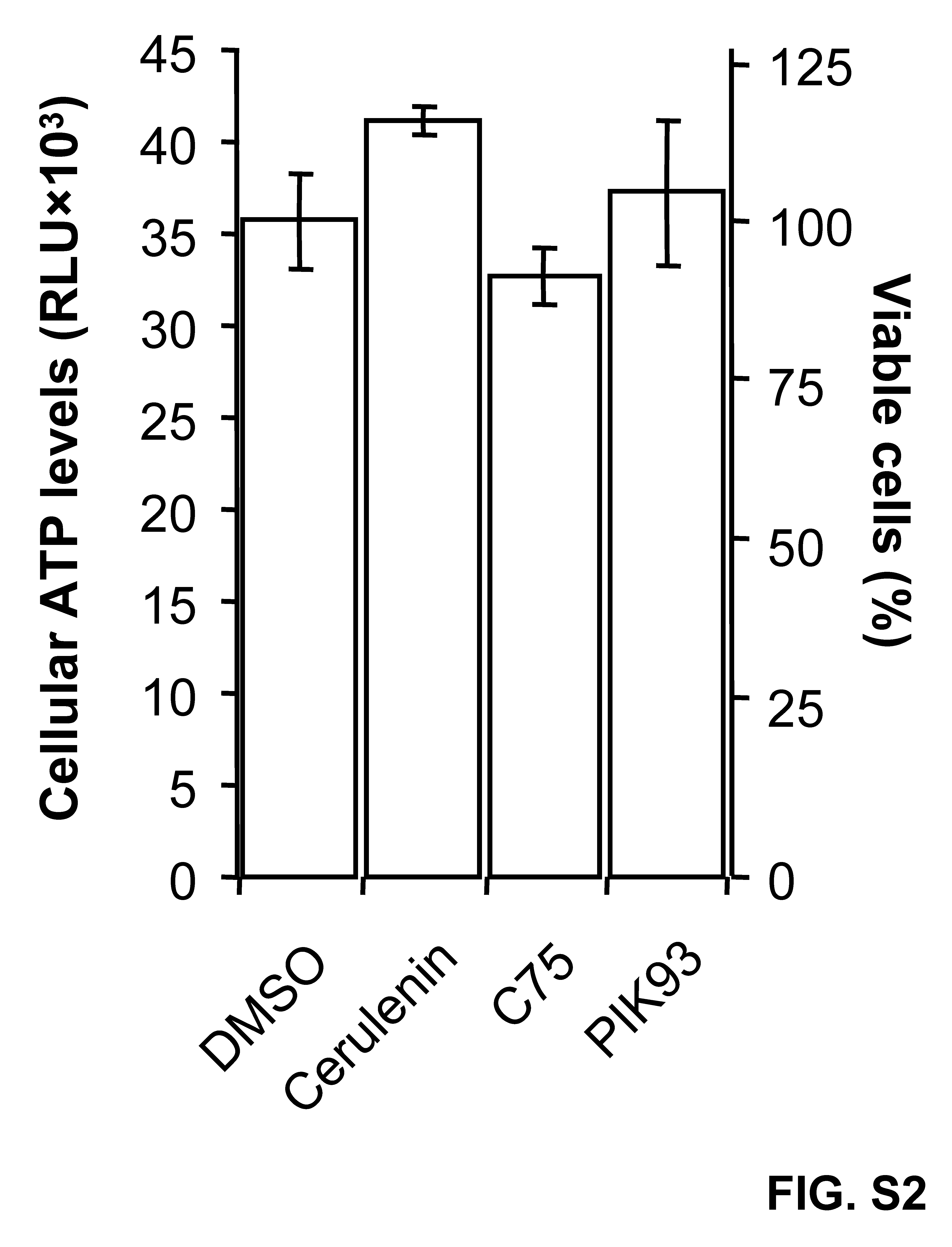

Supplement: Figure S2 — Analysis of cellular viability upon drug treatments. Cellular ATP levels were determined after 24 h of treatment with DMSO (drug vehicle), 15 µM cerulenin, 15 µM C75 or 1 µM PIK93. RLU, relative luciferase units. (TIF) [file pone.0024970.s002.tif]
